# Sequence conservation and combinatorial complexity of *Drosophila *neural precursor cell enhancers

**DOI:** 10.1186/1471-2164-9-371

**Published:** 2008-08-01

**Authors:** Thomas Brody, Wayne Rasband, Kevin Baler, Alexander Kuzin, Mukta Kundu, Ward F Odenwald

**Affiliations:** 1 Neural Cell-Fate Determinants Section, NINDS, NIH, Bethesda, Maryland, USA; 2 Office of Scientific Director, IRP, NIMH, NIH, Bethesda, Maryland, USA

## Abstract

**Background:**

The presence of highly conserved sequences within *cis*-regulatory regions can serve as a valuable starting point for elucidating the basis of enhancer function. This study focuses on regulation of gene expression during the early events of *Drosophila *neural development. We describe the use of *EvoPrinter *and *cis*-Decoder, a suite of interrelated phylogenetic footprinting and alignment programs, to characterize highly conserved sequences that are shared among co-regulating enhancers.

**Results:**

Analysis of *in vivo *characterized enhancers that drive neural precursor gene expression has revealed that they contain clusters of highly conserved sequence blocks (CSBs) made up of shorter shared sequence elements which are present in different combinations and orientations within the different co-regulating enhancers; these elements contain either known consensus transcription factor binding sites or consist of novel sequences that have not been functionally characterized. The CSBs of co-regulated enhancers share a large number of sequence elements, suggesting that a diverse repertoire of transcription factors may interact in a highly combinatorial fashion to coordinately regulate gene expression. We have used information gained from our comparative analysis to discover an enhancer that directs expression of the *nervy *gene in neural precursor cells of the CNS and PNS.

**Conclusion:**

The combined use *EvoPrinter *and *cis*-Decoder has yielded important insights into the combinatorial appearance of fundamental sequence elements required for neural enhancer function. Each of the 30 enhancers examined conformed to a pattern of highly conserved blocks of sequences containing shared constituent elements. These data establish a basis for further analysis and understanding of neural enhancer function.

## Background

Studies over the last two decades have revealed that *cis*-regulatory elements, i.e. enhancers, contain multiple DNA-binding sites for different transcription factors (TFs) that cooperatively function to direct the tissue specific expression of their associated genes [1]. DNA sequence comparisons of different co-regulating enhancers suggest that many of these enhancers rely on different combinations of TFs to achieve coordinate gene regulation [2]. For example, during early *Drosophila *neural development, combinatorial interaction of proneural basic helix-loop-helix (bHLH) TFs with homeodomain proteins, regulate commitment and patterning of neural precursors [3-8].

Cross-species analysis of individual *Drosophila *enhancers, using *EvoPrinter *or conventional alignment based phylogenetic comparative analysis [9,10] and the twelve sequenced *Drosophila *genomes, representing over 160 million years of collective evolutionary divergence, reveals that these enhancers are made up of clusters of highly conserved sequence blocks (CSBs), separated by less conserved sequences of variable length [11]. CSBs that are longer than 8–10 bp are likely to be made up of adjacent or overlapping DNA-binding sites for different TFs. For example, the *Drosophila Krüppel *central domain enhancer contains overlapping highly conserved binding sites for its known regulators [12-14,10]. Specifically, work from the Jäckle laboratory [14] has shown that one CSB of the central domain enhancer, 16 base pairs in length, contains overlapping binding sites for the antagonistic Bicoid activator and the Knirps repressor TFs.

In order to initiate the functional dissection of CSBs that make up neural precursor gene enhancers and to gain a better understanding of their architecture in terms of the substructure of their constituent sequence elements, we have developed a multi-step protocol (collectively known as *cis*-Decoder) that allows for the rapid identification of short 6 to 14 bp DNA elements, termed *cis*-Decoder tags (*c*DTs), within enhancer CSBs; these *c*DTs are shared between CSBs of two or more enhancers with either related or divergent functions [11]. To discover enhancer type-specific elements that regulate gene expression in neural precursor cells – including genes expressed in early delaminating CNS neuroblasts (NBs) and the proneural clusters and sensory organ precursors of the PNS – we have performed *cis*-Decoder analysis of CSBs from *in vivo *characterized enhancers. For early CNS development, we have selected the previously described enhancers of six genes that activate expression in early delaminating CNS NBs: *deadpan *(*dpn*), *hunchback *(*hb*), *nerfin-1*, *scratch *(*scrt*; the SA enhancer), *snail *(*sna*) and *worniu *(*wor*) (Table 1) [15-18]. For the *cis*-regulatory regions that drive expression in the proneural clusters (PNCs) and sensory organ precursors (SOPs) of the PNS we selected the *in vivo *characterized enhancers for *bearded *(*brd*), *deadpan *(*dpn*), *rhomboid *(*rho*), *scrt *and *sna *(Table 1) [19-24].

**Table 1:** *Drosophila *enhancers included in the *cis*-Decoder analysis

**Enhancer**	**# CSBs**	Location*/Size in bp	**References**
**CNS Neuroblast**			
*deadpan*	32	-1405 to -2772/1367	[15]
*hunchback*	26	-36 to -1270/1224	[16]
*nerfin-1*	29	-1529 to -160/1369	A. Kuzin (personal com.)
*scratch*	48	-10867 to -4796/6069	[15]
*worniu*	106	-51 to -6940/5989	[17]
*snail*	26	-244- to -1138/869	[18]
**PNS Precursor Cell**			
*achaete *(DC)	28	-5584 to -7725/2141	[19]
*amos *(3.5)	56	-2397 to +102/2499	[20]
*atonal *(F:2.6)	60	-2807 to +175/2982	[21]
*bearded*	20	-589 to +25/614	[22]
*Pray For Elves*	27	+2236 to +2711/475	[23]
*charlatan*	18	+15440 to +17758/2318	[23]
*deadpan*	18	-4166 to -4677/511	[15]
*ETS-domain lacking*	15	+998 to +2174/1176	[23]
*rhomboid*	21	-6669 to -8419/1720	[23]
*schizo*	20	+31166 to + 32661/1595	[23]
*scute*	7	-2562 to -3015/453	[24]
*scratch*	21	-3898 to -2000/1998	[15]
*Snail*	17	-543 to +40/583	[18]
**E(spl) enhancers**			
*HLHm3*	32	-2176 to +177/2353	[25]
*HLH m5*	38	-1743 to +21/1767	[25]
*HLH m7*	20	-770 to -26/744	[25]
*E(spl)m8*	22	-773 to +188/961	[25]
*HLHmβ*	20	-1012 to +81/1093	[25]
*HLH mγ*	25	-49 to -853/814	[25]
*HLHmδ*	20	-1634 to +25/1659	[25]
*E(spl)m2*	36	-1659 to +48/1707	[26]
*E(spl)m4*	24	-157 to -1056/899	[27,28]
*E(spl)m6*	26	-880 to +7/887	[26]
*E(spl)mγ*	26	-1718 to -40/1678	[26]

*Relative to transcription start site

Our analysis of the CSBs from these characterized enhancers has identified known TF DNA-binding sites and novel sequences of as yet unknown function. Enhancer type-specific sequence elements within CSBs appear in different combinations and contexts in enhancers of co-regulated genes. The information gained from *cis*-Decoder analysis of the neural precursor cell enhancer CSBs was used to discover a novel co-regulating enhancer that directs *Drosophila nervy *expression. Our studies indicate that although specific core DNA-binding sites (such as those for bHLH and homeodomain TFs) are enriched in enhancers of co-regulated genes, enhancer-binding specificity is most likely conferred through sequences that flank the consensus core docking sites. The fact that shared sequence elements of co-regulated enhancers reside in different combinations and positional ordering within each of the enhancers, suggests that their combined presence but not necessarily their relative positions is required for *cis*-regulatory function.

## Results and discussion

### Neural precursor cell enhancers share highly conserved core sequence elements

To determine the extent to which neural precursor cell enhancers share highly conserved sequence elements, we performed *cis*-Decoder analysis of *in vivo *characterized enhancers (Table 1) [15-28]. Our analysis revealed the presence of both novel elements and sequences that contained consensus DNA-binding sites for known regulators of early neurogenesis. Table 2 lists *c*DTs shared by multiple CNS or PNS neural precursor cell enhancers. None of the elements shown were present in our collection of 819 CSBs from *in vivo *characterized mesodermal enhancers, thus ensuring their enrichment in neural enhancers. Highlighted are consensus binding sites for known TFs; basic Helix-Loop Helix (bHLH) factors and Suppressor of Hairless [Su(H)], respectively acting in proneural and neurogenic pathways [7]; Antennapedia class homeodomain proteins [29], identified by their core ATTA binding sequence, and the ubiquitously expressed Pbx- (Pre-B Cell Leukemia TF) class homeodomain protein Extradenticle, a cofactor of many TFs [30], identified by the core binding sequence of ATCA. More than half the conserved *c*DTs were novel, without identified interacting proteins. Many of the CSBs consisted of 8 or more bp, and often contained core sequences identical to binding sites for known factors as well as other core sequences that aligned with shorter novel *c*DTs, suggesting that the longer *c*DTs may contain core recognition sequences for two or more TFs.

**Table 2:** Conserved neural specific sequence elements within two or more neural precursor cell enhancers

CDTs	CNS Neural Precursor Cell Enhancers						PNS Neural Precursor Cell Enhancers												
		
*Gene->*	*dpn*	*hb*	*nf-1*	*scrt*	*sna*	*wor*	*ac*	*amos*	*ato*	*brd*	*char*	*dpn*	*edl*	*pfe*	*rho*	*sc*	*scrt*	*siz*	*sna*
ACT**TGAT**T	1	-	-	1	-	-	-	-	-	-	-	-	-	-	-	-	-	-	-
TTTGA**ATTA**	-	1	-	-	-	2	-	-	-	-	-	-	-	-	-	-	-	-	-
**TAATTGAT**	-	-	-	-	-	2	-	-	-	-	-	-	-	-	-	-	-	-	-
**TGAT**TTCT	-	-	1	-	1	1	-	-	-	-	-	-	-	-	-	-	-	-	-
AA**ATTA**GT	1	-	-	-	-	2	-	-	-	-	-	-	-	-	-	-	-	-	-
AAGTGCAA	-	-	-	2	-	1	-	-	-	-	-	-	-	-	-	-	-	-	-
AA**ATTA**GT	1	-	-	-	-	2	-	-	-	-	-	-	-	-	-	-	-	-	-
A**CAGCTG**T	-	-	1	1	-	-	-	-	-	-	-	-	-	-	-	-	-	-	-
TACGTGT	-	-	-	2	-	1	-	-	-	-	-	-	-	-	-	-	-	-	-
GATTTAC	1	-	-	-	-	2	-	-	-	-	-	-	-	-	-	-	-	-	-
CGGCGTC	-	-	2	-	-	1	-	-	-	-	-	-	-	-	-	-	-	-	-
CAGGATA	-	-	-	2	1	-	-	-	-	-	-	-	-	-	-	-	-	-	-
CACTTCA	1	-	-	-	-	2	-	-	-	-	-	-	-	-	-	-	-	-	-
AATGTGT	2	-	1	-	-	-	-	-	-	-	-	-	-	-	-	-	-	-	-
AATGCAC	-	-	2	-	1	-	-	-	-	-	-	-	-	-	-	-	-	-	-
AACATAA	-	-	1	-	-	2	-	-	-	-	-	-	-	-	-	-	-	-	-
AAAATGC	-	2	-	-	-	1	-	-	-	-	-	-	-	-	-	-	-	-	-
**TGAT**CCA	1	-	-	-	1	-	-	-	-	-	-	-	-	-	-	-	-	-	-
GCACGA	2	-	-	1	-	-	-	-	-	-	-	-	-	-	-	-	-	-	-
GATTCC	-	-	2	1	-	-	-	-	-	-	-	-	-	-	-	-	-	-	-
GAGTGC	-	-	1	1	-	1	-	-	-	-	-	-	-		-	-	-	-	-
ATGGC	-	-	-	2	-	1	-	-	-	-	-	-	-	-	-	-	-	-	-
CTAAGC	1	-	1	1	-	1	-	-	-	-	-	-	-	-	-	-	-	-	-
AATCCC	-	1	-	-	-	3	-	-	-	-	-	-	-	-	-	-	-	-	-
CACCCG	-	-	-	-	2	2	-	-	-	-	-	-	-	-	-	-	-	-	-
AGATAT	1	-	-	-	1	1	-	-	-	-	-	-	-	-	-	-	-	-	-
AGCTTA	-	1	1	-	-	1	-	-	-	-	-	-	-	-	-	-	-	-	-
GGGGCA	1	-	-	1	-	1	-	-	-	-	-	-	-	-	-	-	-	-	-
TTT**TAATTA**	-	-	-	1	-	1	1	-	-	-	-	-	-	-	-	-	-	-	-
CAA**ATTA**G	1	-	-	-	-	2	1	-	-	-	-	-	-	-	-	-	-	-	-
ACAAACAA	-	1	1	-	-	1	-	1	-	-	-	-	-	-	-	-	-	-	-
AGTT**ATTA**	2	-	-	-	-	1	-	-	-	-	-	-	-	-	-	-	-	-	1
TT**TGAT**TT	1	1	-	-	1	-	-	-	-	-	-	-	-	1	-	-	-	-	-
AA**CAGCTG**	-	-	3	1	-	-	-	-	-	-	1	-	-	-	-	-	-	-	-
AAATATG	1	-	1	1	-	1	-	-	1	-	-	-	-	-	-	-	-	-	-
TATTGAA	1	-	-	2	-	-	-	-	-	-	1	-	-	-	-	-	-	-	
ATATTTG	-	-	-	2	-	1	-	-	1	-	-	-	-	-	-	-	-	-	-
AAACTAA	-	2	-	-	-	1	-	-	-	1	-	-	-	-	-	-	-	-	-
GTGTAAA	1	1	-	-	-	1	-	-	-	1	-	-	-	-	-	-	-	-	-
T**TGAT**CC	1	-	-	1	-	1	-	-	-	-		-	-	-	-	-	1	-	-
GGAAAAA	-	-	1	1	-	1	-	-	-	-	-	-	-	-	-	-	-	1	-
CACCCCA	1	-	1	1	-	-	-	-	-	-	1	-	-	-	-	-	-	-	-
CCACCCC	-	1	1	1	-	1	-	-	-	-	2	-	-	-	-	-	-	-	-
ACCCCA	-	-	1	1	1	2	-	-	-	-	1	-	-	-	-	-	-	-	1
**ATTA**GTT	-	-	-	1	-	2	-	-	-	1	-	-	-	-	-	-	-	-	-
AGCTGAC	1	-	1	-	-	1	-	-	-	-	-	-	-	-	-	-	1	-	-
ACAATGA	-	-	1	1	1	-	-	-	1	-	-	-	-	-	-	-	-	-	-
TCCCAC	1	-	-	-	-	1	-	-	-	1	-	-	-	-	-	-	-	-	-
**TTCCCAC**	2	-	-	-	-	-	-	-	-	-	-	1	-	-	-	-	-	-	-
GTTCCCA	1	-	-	-	-	2	-	-	-	-	-	1	-	-	-	-	-	-	-
TCCCAC	2	-	-	-	-	1	-	-	-	1	-	1	-	-	-	-	-	-	-
CC**ATTA**T	-	-	-	1	-	1	-	-	-	-	-	-	-	-	-	-	-	1	-
**TGAT**CC	2	-	-	1	-	1	-	-	-	-	-	-	-	-	-	-	1	-	-
CACGAT	2	-	-	-	-	1	-	-	-	-	-	-	-	-	-	-	1	-	-
ACCTTG	1	-	-	2	-	-	-	-	-	-	1	-	-	-	-	-	-	-	-
CTAAAC	-	1	-	1	1	-	-	-	-	-	-	-	-	-	-	-	-	1	-
G**TGAT**C	-	-	-	1	1	-	-	-	-	-	-	1	-	-	-	-	-	-	-
CACTCA	1	-	-	1	-	1	-	-	-	1	-	-	-	-	-	-	-	-	-
ACCTGA	1	1	-	-	-	1	-	-	-	-	-	-	-	-	-	-	-	-	1
AG**CACGTG**CC	-	-	-	-	-	1	-	-	1	-	-	-	-	-	1	-	-	-	-
CAG**CAGCTG**	-	-	-	2	-	-	-	-	-	-	1	-	-	-	-	-	-	-	-
A**ATTA**GC	-	1	-	-	-	1	2	1	-	-	-	-	-	-	1	-	-	-	-
CGTGCCA	1	-	-	2	-	1	-	1	1	-	-	-	-	-	-	-	-	-	-
TCACACA	1	-	1	-	1	-	-	-	1	-	-	-	1	-	-	-	-	-	-
AAAGTT	1	-	-	1	1	1	-	-	-	-	-	1	-	-	-	-	1	-	-
TCAATAA	1	-	-	1	-	1	-	1	-	-	-	-	-	-	1	-	-	1	-
G**CACTTG**	-	-	-	3	-	-	1	-	-	-	-	-	-	-	-	1	-	-	-
**CACTTG**C	-	-	-	-	-	2	2	-	-	-	-	-	-	-	-	-	-	-	-
GGCTAA	-	1	-	2	1	-	1	1	-	-	-	-	-	-	1	-	-	-	-
CGTGCC	1	-	1	2	-	2	-	1	3	-	-	-	-	-	1	1	-	-	-
CACGTC	-	-	-	11	-	1	-	-	1	1	-	-	-	-	-	-	1	1	-
CAGCTT	-	-	1	-	-	-	-	-	1	-	-	-	-	-	1	-	1	-	-
CAGGTT	1	-	-	1	-	1	-	-	-	-	1	-	-	-	1	-	-	-	1
CGGTT**T**	-	-	-	-	-	1	1	-	1	-	-	-	-	-	-	-	-	1	-
GCTTCC	1	-	-	1	-	1	1	-	1	-	-	-	-	-	1	-	-	-	-
GTTTGA	-	-	-	2	-	2	1	1	1	-	-	-	-	-	-	-	-	-	-
TCACCT	-	-	-	1	1	-	1	-	-	-	-	1	-	-	-	-	1	-	-
AAAAACT	-	-	-	-	-	1	1	-	-	1	-	1	-	-	-	-	-	-	-
AACACGC	-	-	-	-	-	1	1	1	-	-	-	-	-	-	1	-	-	-	-
CAAACAA	-	1	-	-	-	1	-	1	1	-	-	-	-	-	-	-	-	1	-
GTCAATA	1	-	-	-	-	2	-	1	1	-	-	-	-	-	1	-	-	-	-
TG**CAGCTG**	-	-	-	-	-	1	-	-	-	-	1	-	2	1	1	-	-	-	-
CGG**CAGCTG**	-	-	-	1	-	-	-	-	-	-	1	-	1	-	-	-	-	-	-
AATTCATA	1	-	-	-	-	-	-	1	-	-	-	1	-	1	-	-	-	-	-
**ATTA**GCAT	-	-	-	-	-	-	-	1	1	-	-	-	-	-	-	-	-	-	-
AA**ATTA**GC	-	1	-	1	-	1	2	-	-	-	-	-	-	-	-	-	-	-	-
TT**ATTA**CA	-	-	-	1	-	-	-	-	2	-	-	-	-	-	-	-	-	-	-
**TAAT**TGCC	-	-	-	-	-	-	-	-	-	-	-	-	-	-	2	-	-	-	-
G**CAGCTG**T	-	-	-	-	-	-	-	-	-	-	1	-	-	-	1	-	1	-	-
T**ACCTG**G	-	-	-	1	1	-	1	-	-	-	-	-	-	-	-	-	1	-	-
**CACGTG**CT	-	-	-	-	-	-	-	-	1	-	-	-	-	-	1	-	-	-	-
CAAACG	-	-	-	-	-	1	1	1	-	1	-	-	-	-	-	-	-	-	-
CCTACT	-	-	-	-	-	-	-	-	1	-	1	1	-	-	-	-	-	-	-
CCTGTC	-	-	-	-	1	-	-	1	-	-	-	-	-	-	-	-	1	-	1
TGAGAA	-	-	-	-	-	-	1	-	-	-	-	3	-	-	-	-	-	-	-
CGCGAG	-	-	1	-	-	-	-	-	-	-	-	-	-	-	-	-	1	2	-
CGCGTGGCA	-	-	-	-	-	-	-	1	-	-	-	-	-	-	-	1	1	-	-
GCTTTCA**ATTA**	-	-	-	-	-	-	1	-	-	-	-	-	-	-	1	-	-	-	-
**CAGCTG**CAATT	-	-	-	-	-	-	-	-	-	-	-	-	-	1	1	-	-	-	-
G**CACGTG**TGC	-	-	-	-	-	-	-	-	-	1	-	-	-	-	-	-	-	-	1
CAC**CAAATG**G	-	-	-	-	-	-	-	-	1	-	-	-	-	-	-	-	-	-	1
**CACGTG**CAA	-	-	-	-	-	-	-	-	-	1	-	1	-	-	-	-	-	-	-
G**CAGGTG**TA	-	-	-	-	-	-	-	-	1	-	-	-	-	-	-	1	-	-	-
TGGTGGTGG	-	-	-	-	-	-	-	-	-	-	-	2	-	-	-	-	-	-	1
TTGAAAA**A**	-	-	-	-	-	-	-	-	1	-	1	-	-	1	-	-	-	-	-
ATTGCAGC	-	-	-	-	-	-	-	1	-	-	-	-	-	1	1	-	-	-	-
ATTGAAAA	-	-	-	-	-	-	-	-	2	-	1	-	-	-	-	-	-	-	-
GACAACA	-	-	-	-	-	-	-	-	1	1	-	-	-	-	1	-	-	-	-
GAATTGA	-	-	-	-	-	-	1	1	-	-	-	1	-	-	-	-	-	-	-
CTTTCAA	-	-	-	-	-	-	1	2	-	-	1	-	-	-	1	-	-	-	-
GTGAGAA	-	-	-	-	-	-	1	-	-	-	-	2	-	-	-	-	-	-	-
ACGTGTG	-	-	-	-	-	-	1	-	1	1	-	-	-	-	-	-	-	1	1
AACCACC	-	-	-	-	-	-	-	-	-	-	1	1	-	-	-	-	-	-	1
ACCCCTA	-	-	-	-	-	-	-	-	-	1	1	-	-	-	-	1	-	-	-
ACGGAAG	-	-	-	-	-	-	1	-	-	-	-	-	-	-	1	-	1	-	-
AG**ATTA**T	-	-	-	-	-	-	-	1	1	-	-	-	1	-	-	-	-	-	-
AGCGTCA	-	-	-	-	-	-	1	-	-	-	1	-	-	-	-	-	1	-	-
**CATCTG**T	-	-	-	-	-	-	-	-	-	-	-	-	1	-	-	-	1	1	-
CAGCAC	-	-	-	-	-	-	-	-	3	-	-	-	-	-	-	-	3	-	-
GT**AGGA**	-	-	-	-	-	-	-	-	-	-	2	1	-	-	-	-	-	-	-
CCGTGC	-	-	-	-	-	-	-	1	-	-	-	-	-	-	1	-	1	-	-
CGCCTC	-	-	-	-	-	-	-	-	-	-	1	-	-	-	1	-	1	-	-
GAAAGC	-	-	-	-	-	-	1	-	1	-	1	-	-	-	-	1	-	1	-
GAGTCA	-	-	-	-	-	-	-	1	-	1	-	-	-	-	-	-	-	-	1
TAGCCA	-	-	-	-	-	-	1	2	-	1	1	-	-	-	-	-	-	-	-
TCTATT	-	-	-	-	-	-	1	1	-	-	-	-	-	-	-	-	-	-	1
ATCTAA	-	-	-	-	-	-	1	-	2	-	-	-	-	-	-	-	-	-	-

Consensus binding sites for bHLH transcription factors (CANNTG), Antp class homeodomain proteins (ATTA and TAAT), Pbx/Extradenticle (TGAT and ATCA), Tramtrack (AGGA) and Su(H) (TTCCCAC) are bold.

Most *c*DTs discovered in this analysis represent elements that are shared pairwise, i.e., by only two of the NB enhancers examined (see the website for a list of cDTs that are shared by only two of the enhancers examined). The fact that the majority of *c*DTs are shared two ways, with only a small subset of sequences being shared three or more ways, suggests that the *cis*-regulation of early neural precursor genes is carried out by a large number of factors acting combinatorially and/or that many of the identified *c*DTs may in fact represent interlocking sites for multiple factors, and the exact orientation and spacing of these sites may differ among enhancers.

### Neural specific cDTs that contain bHLH TF DNA-binding sites

During *Drosophila *neurogenesis, bHLH proteins function as proneural TFs to initiate neurogenesis in both the central and peripheral nervous system. TFs encoded by the *achaete-scute *complex function in both systems, while the related Atonal bHLH protein functions exclusively in the PNS [31]. Different proneural bHLH TFs, acting together with the ubiquitous dimerization partner Daughterless, bind to distinct E-boxes that contain different core sequences [32]. In addition to the core recognition sequence, flanking bases are important to the DNA binding specificity of bHLH factors [33].

One of the principle observations of this study was that the core central two bases of the hexameric E-box DNA-binding site (CA**NN**TG; core bases are bold throughout) were conserved in all the species used to generate the *EvoPrint*. All of the enhancers included in this study contained one or more conserved bHLH-binding sites (Table 3), with NB and PNS enhancers averaging 3.9 and 4.1 binding sites respectively. More than a third of the core bases in NB bHLH sites contained a core **GC **sequence, and more than a third of the core bases in PNS bHLH sites contained either a core **GC **or a **GG **sequence. The most common E-box among the NB CSBs was CA**GC**TG with 14 sites in four of the six enhancers. The CA**GC**TG and CA**GG**TG E-boxes are high-affinity sites for Achaete/Scute bHLH proteins [22,34]. However the CA**GC**TG site itself is not specific to NB enhancers, as evidenced by its presence in four of the mesodermal enhancer CSBs characterized previously [11]. The most common bHLH-binding site among PNS enhancers was also the CA**GC**TG E-box with 11 occurrences in six of the 13 enhancers. In contrast, the most common bHLH motif in enhancers of the E(spl)-complex [25-28] was CA**AG**TG (data not shown), with 16 occurrences in 8 of the 11 enhancers. CA**GG**TG, previously shown to be an Atonal DNA-binding site [32], was also common in E(spl) enhancers, with 9 occurrences in 8 of the 13 enhancers, but was less prevalent among NB enhancers. The CA**GG**TG box was also overrepresented in PNS and E(spl) enhancers relative to its appearance in NB enhancers, and it was also present in four of the characterized mesodermal enhancer CSBs. The CA**GA**TG box was present six times among PNS enhancers but not at all among NB enhancers. Thus there appears to be some specificity of E-boxes in the different enhancer types. The fact that each of these E-boxes is conserved in all the species in the analysis, suggests that there is a high degree of specificity conferred by the E-box core sequence.

**Table 3:** Conserved bHLH binding sites in NB and PNS enhancer *cis-*Decoder tags

**CANNTG E-box**	**CNS Neural Precursor Cell Enhancers**						**PNS Neural Precursor Cell Enhancers**												
		
	***dpn***	***hb***	***nf-1***	***scrt***	***sna***	***wor***	***ac***	***amos***	***ato***	***brd***	***char***	***dpn***	***edl***	***pfe***	***rho***	***sc***	***scrt***	***siz***	***sna***
CAGCTG	2	-	3	4	-	5	-	-	-	-	3	-	2	2	2	1	1	-	-
CAGGTG	-	-	-	-	1	1	1	-	1	1	1	-	1	-	-	1	2	1	-
CAGATG	-	-	-	-	-	-	-	-	-	-	-	-	1	-	1	-	1	2	1
CAAATG	1	-	-	4	-	2	2	2	1	-	-	-	2	-	-	-	-	-	1
CAATTG	-	-	-	-	-	1	-	-	2	-	-	-	-	-	-	-	-	-	-
CAACTG	-	-	-	1	-	-	-	-	-	-	-	-	-	-	-	-	1	-	-
CAAGTG	-	1	-	3	1	3	2	1	1	-	-	-	-	-	-	-	1	1	-
CATGTG	-	1	-	1	-	-	-	-	-	1	1	-	-	-	-	-	-	-	-
CATATG	-	-	-	-	-	-	-	-	-	-	-	-	-	-	-	-	1	-	-
CACGTG	-	-	1	-	-	2	-	-	2	1	-	1	-	-	1	-	-	-	1

**Figure 1:**
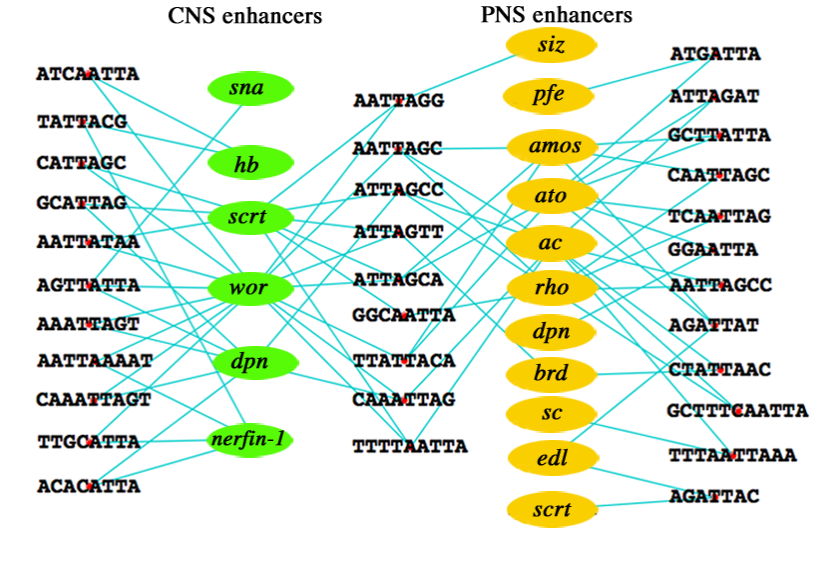
**Shared *c*DTs that contain Antennapedia class homeodomain protein DNA-binding sites within CNS and PNS neural precursor cell enhancers**. Shown is a Cytoscape display of CNS and PNS neural precursor cell enhancer cDTs that contain core ATTA homeodomain DNA-binding sites. *c*DTs flanking the enhancer names are shared by CSBs of a single enhancer type, and *c*DTs positioned between the enhancer names are shared in common by CSBs of the two different enhancer types. Only *c*DTs of 7 or more bases shared by two or more enhancers are portrayed.

Our analysis also reveals that not only are the core bases of E-boxes shared between similarly regulated enhancers, but bases flanking the E-box were also found to be highly conserved and are also frequently shared by these enhancers. Among the E-boxes found in CSBs of NB enhancers (many are illustrated in Table 2) aaCA**GC**TG (core bases of E-box are bold, flanking bases lower case) is repeated three times in *nerfin-1 *and once in *scrt*; gCA**CT**TG is repeated three times in *scrt*; CA**GC**TGCA is repeated twice in *wor*, and CA**GC**TGctg is repeated twice in *scrt *(see Fig 1). In the *dpn *CNS NB enhancer, the E-box CAGCTG is found twice, separated by a single base (CA**GC**TGaCA**GC**TG). None of these sequences were present in mesodermal enhancers examined, but each is found in PNS enhancers; CA**GC**TGCA is repeated multiple times among PNS enhancers. Among the conserved PNS enhancer E-boxes (CA**AA**TGca, gcCA**AA**TG, cacCA**AA**TGg, CA**CA**TGttg, gCA**CG**TGtgc, ttgCA**CG**TG, agCA**CG**TGcc, aCA**GA**TG, ggCA**GA**TGt, CA**GC**TGccg, CA**GC**TGcaattt, gCA**GG**TGta and cCA**GG**TGa) each, including flanking bases, is found in two or three PNS enhancers, and these are distributed among all 13 enhancers. Of these, only agCA**CG**TGcc, CA**GC**TGccg, cCA**GG**TGa were found once in our sample of neuroblast enhancers and none were found in our sample of mesodermal enhancers. The sequence aaCA**AG**TG is found in 4 E(spl) complex enhancers, those for *E(spl)m8, mγ, HLHmδ *and *m6*, and the sequence aCA**GC**TGc is found twice in *E(spl)m8 *and once in *m4 *and *m6; *neither sequence was found in our mesodermal enhancers. Therefore, although a given hexameric sequence may often be shared by all three types of enhancers, NB, PNS and E(spl), when flanking bases are taken into account there appears to be enhancer type-specific enrichment for different E-boxes.

### Neural specific cDTs that contain Antennapedia class homeodomain DNA-binding sites

Antennapedia class homeodomain proteins play essential roles in multiple aspects of neural development including cell proliferation and cell identity [35]. The segmental identity of *Drosophila *NBs is conferred by input from TFs encoded by homeotic loci of the Antennapedia and bithorax complexes [36-38]. For example, ectopic expression of *abd-A*, which specifies the NB6-4a lineage, down-regulates levels of the G1 cyclin, *CycE *[38]. Loss of Polycomb group factors has been shown to lead to aberrant derepression of posterior Hox gene expression in postembryonic NBs, which causes NB death and termination of proliferation in the mutant clones [39].

We have examined the enhancer-type specificity of sequences flanking the Antennapedia class core DNA-binding sequence, ATTA [40]. Nearly 25% of the NB and PNS CSBs examined in this study contain this core recognition sequence. ATTA-containing sites were found multiple times in selected NB and PNS enhancers (Figure 1). The *cis*-Decoder analysis identified 18 different neural specific ATTA containing *c*DTs that were exclusively shared by two or more PNS enhancers or CNS enhancers and 10 were found to be shared between PNS and CNS. The most common *c*DT, **ATTA**gca, was shared by two CNS and two PNS enhancers (Figure 1; consensus homeodomain-binding sites are bold, flanking sequence lower case). In addition, 6 homeodomain-binding site *c*DTs were found twice in *wor *CSBs, a**ATTA**ccg, tttga**ATTA**, aatca**ATTA**, **ATTAAT**ctt and aaacaa**ATTA**g, but not in other CNS or PNS enhancer CSBs. In some cases these *c*DTs were found repeated in given enhancer CSBs. Only one of these *c*DTs aligned with CSBs of enhancers of the E(spl) complex. Given that 2/3 of the occurrences of HOX sites in these promoters can be accounted for by *c*DTs whose flanking sequences are shared between enhancers, it is unlikely that the appearance of these shared sequences occurs by chance.

In summary, the appearance of Hox sites in the context of conserved sequences shared by functionally related enhancers suggests that the specificity of consensus homeodomain-binding sites is conferred by adjacent bases, either through recognition of adjacent bases by the TF itself or in conjunction with one or more co-factors.

### Neural specific cDTs that contain Pbx/Extradenticle sites

Examination of the *c*DTs from *Drosophila *NB and PNS enhancers revealed that many contained the core Pbx/Extradenticle docking site ATGA [41,42]. In *Drosophila*, Extradenticle has been shown to have Hox-dependent and independent functions [43]. Studies have also shown that Pbx factors provide DNA-binding specificity for homeodomain TFs, facilitating specification of distinct structures along the body axis [43]. In the CNS enhancers of *Drosophila*, most predicted Pbx/Extradenticle sites are not, however, found adjacent to Hox sites.

**Figure 2:**
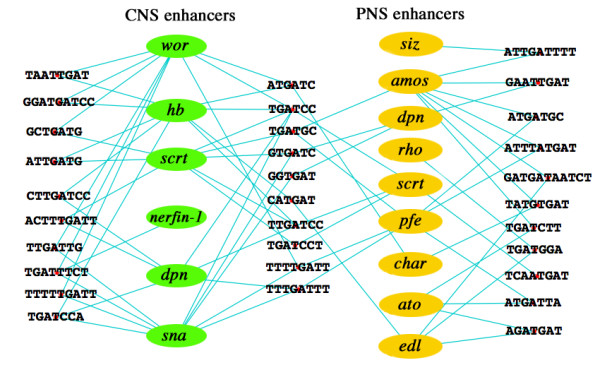
**Shared *c*DTs that contain Pbx/Extradenticle core DNA-binding sites**. Cytoscape analysis of shared Pbx/Extradenticle DNA-binding site (TGAT) containing *c*DT elements present in CNS NB and/or PNS enhancers. *c*DTs flanking the enhancer names are shared by CSBs of a single enhancer type, and *c*DTs positioned between the enhancer names are shared in common by CSBs of the two different enhancer types.

Our analysis revealed that 8 of the Pbx motifs were shared between CNS and PNS enhancer types, and 16 were shared between similarly expressed enhancers (Figure 2), thus indicating that there appears to be some degree of specificity to Pbx site function when flanking bases are taken into account. Three of the Pbx binding-site containing elements also exhibit ATTA Hox sites: 1) the dodecamer GATG**ATTAAT**CT (Pbx site is ATGA, Hox sites in bold) shared by the PNS enhancers *edl *and *amos *(references in Table 1), contains a homeodomain ATTA site that overlaps the Pbx site by a single base, and 2) the smaller heptamer ATG**ATTA**, shared by *pfe *and *ato*, likewise contains a homeodomain ATTA site (bold) that overlaps ATGA Pbx site by a single base. Adjacent Hox and Pbx sites have been documented to facilitate synergy between the two factors [44]. Taken together our findings suggest that, as with homeodomain-binding sites, the conserved bases flanking putative Pbx sites are functionally important. These flanking bases are likely to confer different DNA-binding affinities for Pbx factors or are required for binding of other TFs.

### Neural specific cDTs that contain Suppressor of Hairless binding sites

Also indicating a degree of biological specificity of enhancer types is the distribution of Suppressor of Hairless Su(H) binding sites among neural enhancers. Su(H) is the Notch pathway effector TF of *Drosophila *[45]. The members of the E(spl) complex, both the multiple basic helix-loop-helix (bHLH) repressor genes and the Bearded family members, have been shown to be Su(H) dependent [23,26]. The consensus *in vitro *DNA binding site for Su(H) is RTGRGAR (where R = A or G) [25]. Notch signaling via Su(H) occurs through conserved single or paired sites [46] and the presence of conserved sites for other transcription regulators associated with CSBs containing Su(H) binding sites has been documented [47].

Within the CSBs of the six NB enhancers examined, only two, *dpn *and *wor*, contained conserved putative Su(H)-binding sites; two *dpn *sites matched one of the Su(H) consensus sites (GTGGGAA) and two *wor *sites match the sequence ATGGGAA. Only one of the two *dpn *sites contained flanking bases conforming to the widely distributed CGTGGGAA site of E(spl) Su(H) binding sites and none of the NB enhancers contained paired Su(H) sites typical of the E(spl) enhancers [25,46]. Of the 13 PNS cis-regulatory regions examined, only four enhancers contained putative Su(H)-binding sites [*sna *and *ato *(ATGGGAA), *brd *(GTGGGAG)] and *dpn *(GTGGGAA). *dpn *also contained a pair of sites that conforms to the SPS configuration frequently found in Su(H) enhancers (CSB sequence: AAT**GTGAGAA**AAAAACT**TTCTCAC**GATCACCTT, Su(H) sites in bold, Pbx site is ATCA). The lack of Su(H) sites in PNS enhancers was noted by Reeves and Posakony [23], who suggested that these enhancers are directly regulated by the proneural proteins but not activated in response to Notch-mediated lateral inhibitory signaling. Among the conserved sequences of E(spl) gene enhancers there is an average of 3.4 consensus Su(H) binding sites per enhancer, with most enhancers containing both types of sites, i.e., those with either A or G in the central position (data not shown).

We offer three insights with respect to Su(H) binding sites. First, although *in vitro *DNA-binding studies suggest there is a flexibility in the Su(H) binding site, like the bHLH E-box, comparative analysis shows that within any one the Su(H) sites there is no sequence flexibility. Except for the pair of Su(H) sites in the *dpn *PNS enhancer, none of the CNS or PNS sites contained a central A; less that a quarter of the E(spl) sites consisted of a central A, and all these were conserved across all species examined. In light of the high conservation in these regions the invariant core and flanking sequences are important for the unique Su(H) function at any particular site.

A second finding was the extensive conservation of bases flanking the consensus Su(H) sequence in the E(spl) complex genes (data not shown). For example, the *c*DT **GTGGGAA**ACACACGAC [Su(H) site bold] was present in *HLHm3 *and *HLHm5 *enhancer CSBs, and ACC**GTGGGAA**AC was conserved in *HLHm3 *and *HLHmβ *enhancers. The conservation of bases flanking the consensus Su(H) binding site suggests that the Su(H) site may be flanked by additional binding sites for co-operative or competitive factors, or else, that Su(H) contacts additional bases besides the consensus heptamer.

A third observation is that in most cases Su(H) binding sites are imbedded in larger CSBs, suggesting that CSB function is regulated by the integrated function of multiple TFs. For example the *dpn *NB enhancer Su(H) site is imbedded in a CSB of 24 bases, and the *atonal *PNS enhancer Su(H) site is imbedded in a CSB of 45 bases. In the E(spl) complex, CSB #6 of HLHmγ, consisting of 30 bases and CSB#13 of m8, consisting of 31 bases (each contains a GTGGGAA Su(H) site, a CACGAG element, conforming to a Hairy N-box consensus CACNAG [48,49], and an AGGA Tramtrack (Ttk) DNA-binding core recognition sequence [50], but the order and context of these three sites is different for each enhancer). Although Su(H) binding sites were present in only a minority of NB and PNS enhancers, the conservation of core bases, as well as the complexity of their flanking conserved sequences points to a diversity of Su(H) function and interaction with other factors.

### Neural specific cDTs that contain core DNA-binding sites for other known TFs

Two of these elements, one exclusively present in NB enhancers (C**AGGA**TA) and a second exclusively present in PNS enhancers (GT**AGGA**), contained consensus core AGGA DNA-binding sites for Ttk [50], a BTB domain TF that has been shown to regulate pair rule genes during segmentation and to repress neural cell fates [51-53]. Another site (C**ACCCCA**), shared by both NB and PNS enhancers, conforms to the consensus binding site of IA-1 (ACCCCA), the vertebrate homolog of *nerfin-1 *[54]. Most of the cDTs of Table 2 do not contain sequences corresponding to consensus binding-sites of known regulators of NB expression. The fact that they are represented multiple times in NB CSB sequences suggests that they contain binding sites for unknown regulators of neurogenesis in *Drosophila*.

### Neural-enriched cDTs

Neural enriched *c*DTs that are shared between multiple NB enhancers and also exhibit a low frequency in the sample of mesodermal enhancers examined in this study serve as a resource for understanding enhancer elements that may not have an exclusive neural function [see Additional file 1]. Notable here is the presence of CAGCTG bHLH DNA binding sites (all with flanking A, CC and TC) and Antennapedia class homeobox (Hox) core DNA binding site ATTA [40], as well as additional Ttk and Pbx/Extradenticle sites. Present in this list are portions of sequences conforming to Su(H) binding sites described above. Of particular interest in this table are sequences that are also enriched in the PNS (p); these sites may bind factors that play similar developmental roles in different tissues. For example, the presumptive Ttk site, AA**AGGA **(core sequence in bold) is highly enriched in segmental enhancers. Thus, some of these sites can be identified as targets of known TFs, but the identity of most are as yet unknown. These elements shared by multiple enhancers may be useful in identifying other enhancers driving expression in NBs.

### *cis*-Decoder analysis reveals a complex sub-structure of enhancer CSBs

*EvoPrint *analysis revealed that all of the enhancer regions examined in this study contained multiple CSBs that were greater that 15 to 20 bases in length. The occurrence of overlapping DNA-binding sites for different TFs is currently the best explanation for the maintenance of intact CSB sequences across ~160 millions of years of collective species divergence. Our analysis has revealed that the sequence context, order and orientation of shared *c*DTs can differ between co-regulating enhancers.

**Figure 3:**
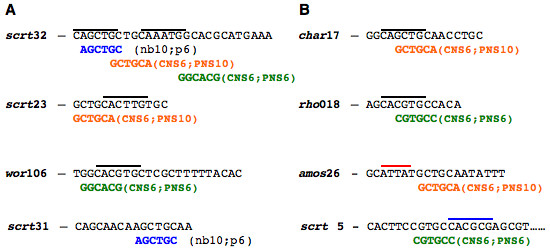
**Shared sequence elements are found in different orientations and patterns within CSBs of neural precursor cell enhancers**. Shown are CSBs from CNS (A) and PNS (B) enhancers aligned to three different frequently found neural specific or enriched *c*DTs. Shown in parentheses is the number of appearances of each *c*DT among CNS NB enhancers (nb), and PNS enhancers (p). Putative bHLH, Hox and Hairy TFDNA-binding sites are over-lined black, red or blue, respectively.

Two examples are given here of the complex contextual appearance of *c*DTs that appear frequently in CNS and PNS enhancers (Figure 3). Each of the eight CSBs shown was nearly fully 'covered' by *c*DTs of the NB library (data not shown), suggesting that each contains multiple overlapping binding sites for a number of TFs. First, examination of the distribution of *c*DT GCTGCA reveals that it overlaps, by one and two bases, adjacent but different consensus bHLH sites in *scrt *CSB^#^32, while in *scrt *CSB^#^23 it overlaps a third consensus bHLH sequence by two bases. In the PNS enhancer *char*, in CSB^#^17, GCTGCA overlaps a bHLH site, but in a different configuration (overlapping four bases) than found in the two CNS enhancers illustrated in Figure 3A. In *amos *CSB^#^26, GCTGCA appears adjacent to a HOX site and does not overlap a bHLH site. Second, examination of the distribution of the *c*DT GGCACG reveals that it overlaps different consensus bHLH sites in *scrt *CSB^#^32 and *wor *CSB^#^106, overlapping the bHLH site in the former by one base and in the latter by four bases. GGCACG overlaps a CAGCTG bHLH-binding site in *rho *CSB^#^18, but in a different configuration than the overlap with CAGCTG in the *wor *CSB. In the PNS enhancer *scrt*, GGCACG in CSB^#^5 overlaps a Hairy site N-box (consensus CACNAG) [48,49]. N-boxes were most common in E(spl) CSBs, but were also present in NB and PNS enhancer CSBs. In these two examples, and others we have examined, there is no consistent spatial constraints to the association of known TF-binding sites (i.e., bHLH-binding E-box sites) with novel *c*DTs; a picture that emerges is one of combinatorial complexity, in which known or novel *c*DTs are associated with each other in different contexts on different CSBs.

As an initial step toward determining if different TFs interacted with one another or competed for flanking DNA-binding sites, we examined the proximity of known binding sites to one another in CSBs for bHLH, Hox, Pbx and Su(H). The results of this analysis for NB CSBs are shown in Table 4; data for other enhancer types is summarized here. Most striking was the presence of multiple adjacent Hox ATTA sites (10 instances on NB CSBs) and combinations of Hox and Pbx sites (9 instances NB CSBs). A typical example is the association of one Pbx site, a bHLH site and two Hox sites on a *wor *NB enhancer CSB (AAT**CATTTG**TAATAATTAG; Pbx site is ATCA, Hox sites are TAAT and ATTA, and bHLH site is bold). Associations of Hox and Pbx sites was also apparent in PNS enhancer CSBs, and in addition there was a high level of combined Hox and bHLH sites (11 instances on PNS CSBs), but in E(spl) enhancers only a higher level of the combination of Hox and Pbx sites (8 instances) was apparent. An example of the association of Hox and bHLH sites in a PNS enhancer is found in an *achaete-scute *dorso-central enhancer CSB (CAAAACAA**CACTTG**CTCTATTAAC; bHLH site in bold and Hox site is ATTA). There was also a distinctly higher level of Pbx sites on the same CSBs as bHLH sites in NBs CSBs (6 instances), but this combination was not apparent for PNS or E(spl) CSBs. Association of bHLH sites with Su(H) binding sites was apparent in E(spl) enhancer CSBs, especially when presence on adjacent CSBs (14 instances) was taken into account. Only in one of the 7 instances of paired Su(H) sites on E(spl) enhancers were these sites on the same CSBs, while in four other instances they were on adjacent CSBs. Although we often find sites in close proximity, both known and functionally uncharacterized sites are, with a few exceptions, not present in fixed uniform orientation in similarly regulated enhancers. This highlights the complex combinatorial arrangement and position flexibility of TF-binding sites within enhancer CSBs.

**Table 4:** Co-appearance of TF binding sites within CSBs and in adjacent CSBs.

	Pbx	bHLH	Hox	Su(H)
Pbx	5/6*	-	-	-
bHLH	6/12	2/4	-	-
Hox	9/14	3/12	10/23	-
Su(H)	0/2	0/0	1/3	0/0
Total	47	36	57	3

*First and second numbers represent appearance on same and adjacent CSBs, respectively.

### The use of *cis-Decoder*, *FlyEnhancer *and *EvoPrinter *to identify novel enhancers

We have used the information derived from *cis-Decoder *analysis of neural precursor cell enhancers to search for other genomic sequences with similar *cis*-regulatory properties. Having identified *c*DTs found multiple times among NB enhancers, we used the genomic search tool *FlyEnhancer *[55] to identify *Drosophila melanogaster *genomic sequences that contained clusters of the following *c*DTs (number in parenthesis is the total number of each *c*DT in our sample of six NB enhancers): GGCACG (6), GGAATC (4), TGACAG (6), TGGGGT (4), CAGCTG (14), TGATTT (9) CAAGTG (7), CATATTT (5), TGATCC (7) and CTAAGC (6). As a lower limit, a minimum of three CAGCTG bHLH sites was set for this search, because of the prevalence of this site in *nerfin-1 *and *deadpan *NB enhancers. Each sequence detected by this search was subjected to *EvoPrinter *analysis to determine the extent of its sequence conservation. Among the *c*DT clusters identified, our search identified a 5' region adjacent to the *nervy *gene ([] that contained three conserved CAGCTG sites as well five other sites identical to TGACAG, GGAATC, TGGGGT, GGCACG and CATATTT (see below). *nervy*, originally identified as a target of homeotic gene regulation, is expressed in a subset of early CNS NBs, as well as in PNS SOP cells [56]. Later studies have implicated *nervy*, along with cyclic adenosine monophosphate (cAMP)-dependent protein kinase (PKA) in antagonizing Sema-1a-PlexA-mediated axonal repulsion [57], and *nervy *has been shown to promote mechanosensory organ development by enhancing Notch signaling [58].

**Figure 4:**
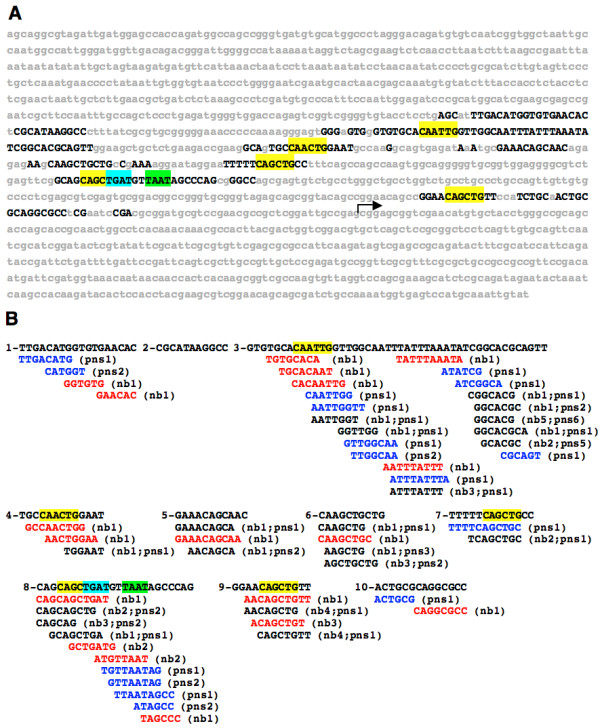
***EvoPrinter *and *cis*-Decoder analysis of the *Drosophila nervy *neural precursor cell enhancer region**. *EvoPrinter *(A) and *cis*-Decoder (B) analysis of a *nervy *neural precursor cell enhancer positioned 90 bp upstream from the *nervy *transcriptional start site. A) Shown is an *EvoPrint *of the *Drosophila melanogaster nervy *gene 5' flanking DNA (487 bp). The predicted *nervy *transcriptional start site is denoted with an arrow. Test species included in the comparative analysis were *D. simulans, D. sechellia, D. erecta, D. yakuba, D. ananassae, D. persimilis, D. pseudoobscura, D. willistoni *and *D. grimshawi*. Black capital letters represent bases conserved in all, or all but one, species. Putative TFDNA-binding sites within the conserved sequences are highlighted (bHLH E-box sites, yellow; an Pbx/Extradenticle site, blue; and an Antennapedia class homeodomain binding site, green). B) Conserved sequence blocks identified in the *nervy EvoPrint *(A) were extracted and scanned for the presence of neural precursor cell enhancer *c*DTs. *c*DTs were generated using NB and PNS enhancer CSBs listed in Table 1. *c*DTs generated by the inclusion of the *nervy *CSBs in the *c*DT library construction are also shown. CNS neuroblast specific cDTs are highlighted in red typeface, PNS precursor cell specific are noted with blue typeface and those present in both are indicated with black typeface (the number of enhancers that contain a *c*DT is also indicated).

**Figure 5:**
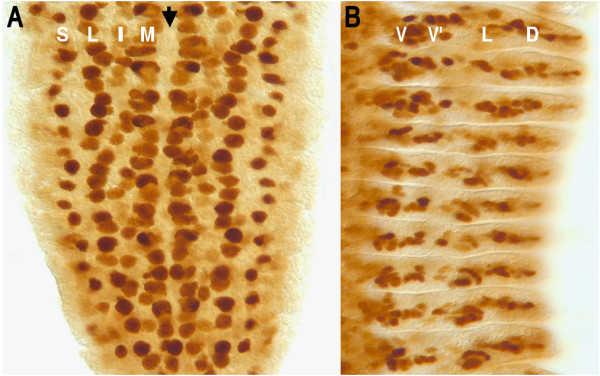
**Expression pattern of the *nervy *enhancer-GFP reporter transgene during embryonic CNS and PNS development**. Shown are GFP immunostains of stage 10 (A) and stage 13 (B) embryos from a transformant line that contains the *nervy *upstream genomic sequence (shown in Figure 4A) adjacent to a minimal promoter/GFP reporter transgene (anterior is up). A) During early nervous system development, GFP reporter expression is detected in CNS neuroblasts and in PNS sensory organ precursor cells. The letter S indicates the PNS sensory organ precursor column and letters L, I, and M mark the lateral, intermediate and medial CNS neuroblast columns, respectively. Arrow indicates the ventral cord midline. B) GFP reporter expression is also detected in the secondary precursor cells of the developing PNS. Shown is the right half of the thoracic and abdominal segments. The letters V, V', L and D indicate the ventral, lateral and dorsal PNS neuronal cell clusters [59].

*EvoPrinter *analysis revealed that the cluster of neural precursor cell enhancer *c*DTs positioned 90 bp upstream from the *nervy *transcribed sequence contains highly conserved sequences (Figure 4A; chr2R:20,162,556-20,163,290). This region contains 10 CSBs that include six conserved E-boxes, three of which conform to the CAGCTG sequence that was prominent in *nerfin-1 *and *deadpan *promoters. To determine if this region functions as a neural precursor cell enhancer, we generated transformant lines containing the *nervy *CSB cluster linked to a minimal promoter/GFP reporter transgene (see methods section). Our analysis of the reporter expression driven by the *nervy *upstream fragment revealed a pattern indistinguishable from early *nervy *mRNA expression [56] (Figure 5). Specifically, we detected expression in a large subset of early delaminating NBs and in SOPs and secondary precursor cells of the PNS. Significantly, the *nervy *enhancer, unlike *nerfin-1 *and *deadpan *NB enhancers, activates reporter expression in then PNS and not just in early NBs.

A new *c*DT-library was generated combining the *nervy *enhancer CSBs and the NB and PNS enhancer CSBs used to generate the libraries described above. The new *c*DTs, along with the previously defined *c*DTs were aligned back to *nervy *CSBs (Figure 4b). Most *c*DTs were found only once in previously examined NB or PNS CSBs, but 21 cDTs appeared in our original analysis, described above, that did not include the *nervy *enhancer. The addition of this new enhancer to our analysis resulted in the discovery of a significant number of *c*DTs that had not been found previously. Three *c*DTs that were identified in the previous analysis, tCA**GC**TGc, cagCA**GC**TG and aaCA**GC**TG, contain bHLH DNA-binding sites (central bases of E-box in bold, flanking sequence are lower case). Aligning *c*DTs that are specific to the CNS or PNS may indicate sequences required to specifically drive expression in either the CNS or PNS.

## Conclusion

The major finding of this study is that enhancers of co-regulated genes in neural precursor cells possess complex combinatorial arrangements of highly conserved *c*DT elements. Comparisons between NB and PNS enhancers identified CNS and PNS type-specific *c*DTs and *c*DTs that were enriched in one or another enhancer type. *cis*-Decoder analysis also revealed that many of the conserved sequences contain DNA-binding sites for classical regulators of neurogenesis, including bHLH, Hox, Pbx, and Su(H) factors. Although *in vitro *DNA-binding studies have shown that many of these factors have a certain degree of flexibility in the sequences to which they bind, defined in terms of a position weight matrix [60], our studies show that for any given appearance these sites are actually highly conserved across all species of the *Drosophila *genus. The genus invariant conservation in many of these characterized binding sites indicates that there are distinct constraints to that sequence in terms of its function.

The high degree of conservation displayed in the enhancer CSBs could derive from unique sequence requirements of individual TFs, or the intertwined nature of multiple DNA-binding sites for different TFs. Thus there is a higher degree of biological specificity to these sites than the flexibility that is detected using *in vitro *DNA-binding studies. As an example, the requirement for a specific core for the bHLH binding site, i.e., for a CAGCTG E-box for *nerfin-1*, *deadpan *and *nervy*, suggests that it is the TF itself that demands sequence conservation; however, the requirement for conserved flanking sequences suggests that additional specific factors may be involved. Although the inter-species conservation of core and flanking sites has been noted by others [25], the extent of this conservation is rather surprising. To what extent and how evolutionary changes in enhancer function take place, given the conservation of core enhancer sequences, remains a question for future investigation.

In addition to classic regulators of neurogenesis, *cis*-Decoder reveals additional conserved novel elements that are widely distributed or only detected in pairs of enhancers. Many of these novel elements flank known transcription binding motifs in one CSB, but appear independent of known motifs in another. The appearance of novel elements in multiple contexts suggests that they may represent DNA-binding sites for additional factors that are essential for enhancer function. Only through discovery of the factors binding these sequences will it become clear what role they play in enhancer function.

Preliminary functional analysis of CSBs within the *nerfin-1 *neuroblast enhancer reveals that CSBs carry out different regulatory roles (Alexander Kuzin, unpublished results). Altering *c*DT sequences within the *nerfin-1 *CSBs reveals that most are required for cell-specific activation or repression or for normal enhancer expression levels. CSB swapping studies reveals that, for the most part, the order and arrangement of a number of tested CSBs was not important for enhancer function in reporter studies. The discovery of the *nervy *neural enhancer by searching the genome with commonly occurring NB *c*DTs underscores the potential use of *EvoPrinter *and *cis*-Decoder analysis for the identification of additional neural enhancers. By starting with known enhancers and building *c*DT libraries from their CSBs, one now has the ability to search for other genes expressed during any biological event.

## Methods

### Generation of *EvoPrints *and CSB-libraries

*EvoPrinter *analysis was performed as described [10,61]. This analysis used *EvoPrinterHD *(please see Availability & requirements for more information) a second-generation *EvoPrinter *program that uses an *enhanced*-BLAT algorithm for increased resolution of conserved sequences [61]. Detailed instructions are provided at the *EvoPrinter *web site.

When possible, all twelve *Drosophila *species were used for the *EvoPrint *analysis, while species that exhibited sequencing gaps were excluded. CSBs within enhancers were curated from either an *EvoPrint*, which reveals bases conserved in all species, or a relaxed print (also known as an *EvoDifference *profile) that identifies base pairs that are conserved in all but one of the species. The collective evolutionary divergence for all of the *EvoPrints *was greater than 140 My and in most cases, when all twelve species were included in the analysis, *EvoPrints *represented over ~200 My of additive divergence. With the exception of two NB enhancers, *scrt *and *wor*, the size of each curated sequence was less than 1800 bases (Table 1). CSBs of 6 bp or longer were extracted from the EvoPrints using EvoPrint parser to generate CSB libraries. The number of CSBs in each enhancer, enhancer length, and relation of the enhancer with respect to the transcriptional start site is shown in Table 1. Lists of CSBs for each library are given at the *cis*-Decoder web site (please see Availability & requirements for more information).

### Generation of *cis*-Decoder Tag libraries

In order to focus the analysis on neural-specific and neural-enriched *c*DTs, those cDTs that were found at high frequency in non-neural (mesodermal) enhancers were placed in a shared/common *c*DT-library. To identify neural specific *c*DT elements, the frequency of *c*DTs was scored against an out-group of mesodermal CSBs [11], and subsequently the common elements were removed. Prior to removal of mesodermal *c*DTs, the number of NB *c*DTs was 856, whereas after removal of shared *c*DTs, the number dropped to 272, indicating that the majority of *c*DTs shared by NB enhancers were also present in mesodermal enhancers.

Three *c*DT-libraries were generated by alignment of NB, PNS and E(spl) CSBs and are provided at the cis-Decoder web site (please see Availability & requirements for more information). The number of *c*DTs in each library was 272, 333 and 226 respectively. Of the 272 NB *c*DTs, less than half (120) aligned exclusively with NB CSBs, and did not align with PNS or E(spl) CSB sequences. Only 21% of the NB *c*DTs corresponded to PNS tags – in other words only 21% of the NB tags aligned two times or more with PNS CSBs.

### Cytoscape analysis

We have adapted the biomolecular interaction network software Cytoscape [62] in order to display shared *c*DTs from different enhancer CSBs. The following data structure was used: node1 xx node2, where node1 is the name of an enhancer, xx refers to any designator and node2 is the *c*DT sequence. This data structure facilitates the display of enhancer identity and shared sequence elements in an interactive pattern. Cytoscape analysis requires elimination of the reverse complements of *c*DTs in order to avoid duplicate representation. To eliminate duplicate reverse-complement *c*DTs, we used the program *c*DT-Uncomplementer (please see Availability & requirements for more information). After removing duplicates, *c*DT-cataloger was used to name each node according to the enhancer aligning with that *c*DT.

### Identification of novel neural precursor cell enhancers

To identify novel enhancers that direct gene expression in neural precursor cells, we curated *c*DTs that were shared by multiple identified NB enhancers and submitted them to the web-based genomic search tool *FlyEnhancer *[55], to discover other genomic regions with similar densities of *c*DTs. Candidate sequences that contained densities of *c*DTs alignments were subject to *EvoPrinterHD *analysis to determine the extent of conservation. Candidate enhancer regions were selected for enhancer/reporter studies.

### Generation and analysis of *nervy *enhancer/reporter transformant lines

Genomic DNA containing the putative *nervy *enhancer (734 bp) was amplified by PCR using standard methods. Primers for the *nervy *upstream region including BglII and Nhe1 sites (bold) were respectively **AGATCT**CTAAAGCCCTCGATGTGCCC (5') and **GCTAGC**TCCGACCAGTCGTAAGTGGCG (3'). Fragments were gel purified and cloned into the pCRII-TOPO double promoter vector. Sequencing verified the fidelity of the PCR and cloning. After cutting with Bgl and Nhe1, gel purification was performed and fragments were cloned into pH-Stinger [63]. Details of our procedure are available upon request. The generation of transformant lines and embryo immunohistochemistry were carried out as described previously [64].

## Availability & requirements

*EvoPrinterHD*: 

*cis*-Decoder, CSB-libraries: 

*cis*-Decoder, cDT-libraries: 

*c*DT-Uncomplementer: 

## Authors' contributions

WR and KB participated in the design and implementation of the algorithms. AK and MK participated in the cloning of enhancers. TB and WFO conceived of the study, participated in the design and coordination of the algorithms and prepared the manuscript. All authors have read and approved the final draft of the manuscript.

## Supplementary Material

Additional file 1 *cis*-Decoder tags with multiple hits on two or more NB enhancers. All are NB enriched with a low level of hits on mesoderm CSBs. Click here for file
