# Supplementary material for: Sequence conservation and combinatorial complexity of Drosophila neural precursor cell enhancers
Source: BMC Genomics. 2008 Aug 1;9:371. doi: 10.1186/1471-2164-9-371 (PMC2529316; doi:10.1186/1471-2164-9-371)
Supplement: Additional file 1 — cis-Decoder tags with multiple hits on two or more NB enhancers. All are NB enriched with a low level of hits on mesoderm CSBs. [file 1471-2164-9-371-S1.doc]

**Supplemental Table 1 - *cis*-Decoder tags with multiple hits on two or more NB enhancers. All are NB enriched (with low level of hit on mesoderm library)**

| **n6;p7-AAAAAC** | **hb wor scrt3 hb |* char rho2 ac dpn brd siz** | **n4;p1-GCACAA** | **scrt2 sna hb |dpn** |
| --- | --- | --- | --- |
| **n4;p2-AAAAAG** | **nerfin2 sna wor |ato char** | **n3;p5-GCAGCA** | **wor nerfin scrt |amos scrt ato char rho** |
| **n3;p4-AAAAGG** | **scrt dpn wor |dpn siz brd ac** | **n3;p1-GCCTCA** | **wor nerfin wor |scrt** |
| **n4;p2-AAAGCC** | **sna hb nerfin2 |ato char** | **n4-GCCGCA** | **wor2 scrt nerfin** |
| **n7;p2-AAAGGA**** | **nerfin3 scrt2 wor sna |edl siz** | **n4;p2-GCTGAC** | **nerfin wor dpn |ato scrt** |
| **n3;p4-AACAAC** | **nerfin2 wor |sna2 ac char** | **n5;p10-GCTGCA** | **scrt3 wor2 |ato edl2 pre2 rho2 sc char amos** |
| **n3;p4-AACAAG** | **wor scrt2 |ato2 siz scrt** | **n5;p6-GCTGCC** | **wor scrt2 wor3 |edl rho ac ato char2** |
| **n4;p4-AACACG** | **scrt3 wor |siz ac amos rho** | **n5;p3-GCTGTT** | **nerfin3 scrt2 |char scrt pfe** |
| **n4;p1-AACATA** | **wor nerfin wor dpn |ato** | **n4;p2-GGCGCA** | **wor dpn wor hb |sna rho** |
| **n6;p3-AACTAA** | **scrt2 hb2 wor2 |brd siz ato** | **n4;p24-GGGAAA** | **dpn wor2 scrt |sna2** |
| **n3;p1-AAGCAC** | **hb nerfin scrt |rho** | **n3-GGGTGA** | **nerfin wor2** |
| **n4;p3-AAGCCA** | **dpn sna wor hb |ac amos scrt** | **n3;p3-GTAATA** | **nerfin wor hb |amos ato sna** |
| **n3;p2-AAGTGA** | **wor3 |ac brd** | **n3;p2-TAACAA** | **nerfin scrt wor |brd ato** |
| **n5;p3-AATAAC** | **dpn3 wor2 |sna ac2** | **n3;p2-TAGAAA** | **Scrt2 wor |char amos** |
| **n5;p2-AATATG** | **scrt wor dpn nerfin2 |scrt ato** | **n6;p5-TATTGA** | **scrt2 dpn2 sna wor |ato amos rho char sc** |
| **n3;p2-AATCCA** | **wor2 |dpn rho** | **n3;p2-TGCCAA** | **scrt3 |scrt ato** |
| **n4;p4-ACACGC** | **wor3 scrt |amos ato ac rho** | **n3;p3-TGCTAA** | **scrt nerfin wor |amos ato2** |
| **n4;p2-ACAGGA** | **sna scrt3 |scrt char** | **n7;p3-TGGGAA** | **wor dpn2 scrt wor3 |sna dpn ato** |
| **n7;p1-ACATAA** | **nerfin2 wor4 sna |amos** | **n3;p1-AAATAGC** | **nerfin scrt2 |amos** |
| **n3;p2-ACGACT** | **hb nerfin dpn |ato edl** | **n4;p1-AAATGAA** | **wor2 dpn scrt |pfe** |
| **n5-ACGCCG** | **nerfin2 wor2 scrt** | **n4;p2-AAATTAG** | **dpn wor3 |ac2** |
| **n4-ACGTGA** | **wor3 scrt** | **n3-AAATGCG** | **hb nerfin wor** |
| **n6;p8-ACGTGC** | **wor4 nerfin2 |dpn rho2 ato3 sna** | **n5-AACAAAT** | **wor4 scrt** |
| **n5;p7-ACGTGT** | **wor2 scrt3 |ac siz2 ato sna brd2** | **n5;p1-AACAATG** | **scrt nerfin2 wor sna |ato** |
| **n4-ACTTCA** | **dpn wor2 scrt** | **n4-AACTGCA** | **dpn2 wor hb** |
| **n4;p1-AGAAAT** | **wor scrt sna wor |amos** | **n6;p2-AAGTGCA** | **scrt3 wor2 nerfin |ac dpn** |
| **n3;p2-AGCAAC** | **scrt2 wor |sna amos** | **n3;p1-AATAAAA** | **dpn wor2 |ato** |
| **n4;p1-AGCAAT** | **wor2 sna nerfin |rho** | **n6;p2-AATCAAA** | **scrt sna hb2 dpn |pfe2** |
| **n3;p1-AGCACT** | **scrt3 |scrt** | **n3;p2-AATCATT** | **nerfin scrt wor |pfe ato** |
| **n3-AGCTAA** | **wor2 dpn |** | **n4;p1-AATGAAT** | **sna scrt wor dpn |ato** |
| **n5;p2-AGCTGA** | **scrt wor2 nerfin dpn |sc scrt** | **n4;p1-AATGCGC** | **scrt hb wor2 |edl** |
| **n4;p4-AGGACA** | **scrt nerfin2 sna |scrt brd ato dpn** | **n3-AATTCAA** | **wor2 scrt |** |
| **n3;p3-AGGTAA** | **Dpn scrt2 |ato char2** | **n4;p5-ACAAACA** | **wor2 nerfin hb |dpn char ac amos2** |
| **n4;p3-AGGTAG** | **sna wor2 hb |brd2 amos** | **n3-ACACGTA** | **scrt2 wor** |
| **n7;p3-AGTGCA** | **scrt3 wor2 nerfin2 |scrt ac dpn** | **n7;p3-ACAGCTG** | **nerfin2 scrt3 dpn |rho scrt char** |
| **n9;p16-AGCTGC** | **wor4 scrt4 dpn |pfe3 char4 rho3 edl4 scrt sc** | **n5;p1-ACATAAA** | **wor4 nerfin |amos** |
| **n4-AGTTTG** | **hb scrt wor sna** | **n3;p1-ACTTGCA** | **wor2 scrt |ac** |
| **n3;p2-ATACAA** | **nerfin scrt2 |siz sna** | **n4-ACTTTGA** | **dpn hb wor scrt** |
| **n3;p2-ATAGAA** | **dpn scrt2 |char sna** | **n4;p1-ATCAACA** | **dpn2 scrt2 |scrt** |
| **n3;p1-ATCAAG** | **hb dpn scrt |brd** | **n4;p2-ATGAAAA** | **wor dpn hb wor |ato2** |
| **n3;p1-ATCGTG** | **dpn2 wor |dpn** | **n3;p1-ATGTGTG** | **dpn nerfin sna |edl** |
| **n3;p2-ATGAAG** | **wor2 scrt |dpn pfe** | **n3-ATTCAAT** | **scrt wor dpn** |
| **n4;p2-ATGCAC** | **nerfin3 sna |dpn ac** | **n4;p6-ATTGACA** | **dpn sna wor2 |rho2 ato2 ac amos** |
| **n3;p2-ATGGGA** | **wor3 |ato sna** | **n3;p1-ATTTACA** | **dpn wor2 |brd** |
| **n5;p7-ATTAGC** | **scrt2 dpn wor2 |amos rho ato2 siz ac2** | **n3-CAATCAA** | **wor2 sna** |
| **n4;p4-ATTTGG** | **scrt3 hb |amos edl sna ato** | **n3;p1-CAATGAA** | **sna scrt2 |ato** |
| **n7;p9-CAAACA** | **wor4 scrt nerfin hb |ato2 edl ac dpn amos2 siz char** | **n4;p1-CAGCTGA** | **dpn wor nerfin scrt |sc** |
| **n3;p1-CAAAGC** | **scrt nerfin hb edl|** | **n3;p2-CAGGTAG** | **hb wor sna |brd** |
| **n6;p1-CAAATA** | **wor3 scrt3 |ato** | **n3;p2-CATGAAA** | **scrt dpn nerfin |ato2** |
| **n5;p3-CAGGAA** | **scrt4 hb |scrt2 char** | **n3;p1-CCCATGA** | **wor3 |ato** |
| **n3;p2-CAGTGG** | **wor2 nerfin |brd ato** | **n3-CGTGACA** | **wor3** |
| **n5;p3-CATGAA** | **nerfin wor scrt dpn wor |ato2 dpn** | **n6;p1-GAAATCA** | **nerfin2 wor2 scrt sna |ato** |
| **n4;p7-CCACCA** | **wor2 sna nerfin |dpn3 sna** | **n4;p1-GCGCATT** | **scrt wor2 hb |edl** |
| **n6;p3-CCACCC** | **nerfin scrt2 wor2 hb |char2 ato** | **n3;p2-GCTGACA** | **dpn wor2 |scrt ato** |
| **n4;p3-CCTTGA** | **sna scrt nerfin |char2 brd** | **n4-GTGACAA** | **wor4** |
| **n3;p2-CCTTTC** | **nerfin2 wor |amo2** | **n4-GTTGCCA** | **dpn scrt nerfin2** |
| **n3;p2-CGAAAC** | **nerfin sna wor |siz char** | **n5;p3-TAATTGA** | **hb wor4 |ac rho ato** |
| **n3;p2-CGAAAG** | **scrt2 wor |amo2** | **n3-AAAACATT** | **sna wor hb** |
| **n3-CGTTCA** | **wor3** | **n3;p2-AAACAAAC** | **wor2 sna |char amos** |
| **n4;p6-CTGCAA** | **wor scrt2 hb |edl2 rho amos char pfe** | **n3-AAATGAAT** | **wor scrt dpn** |
| **n3;p2-CGACAG** | **wor2 nerfin |rho edl** | **n3;p1-ATGATTTC** | **wor2 scrt |ato** |
| **n3;p2-CGGCAG** | **wor2 nerfin |edl char** | **n4;p1-ATAAAAAT** | **dpn2 wor dpn |ato** |
| **n4;p6-CTTTCA** | **nerfin2 scrt wor |rho char amos2 ato ac** | **n3;p4-CAGCTGCC** | **wor2 scrt |edl rho char2** |
| **n3;p1-GAAACC** | **nerfin sna dpn |char** | **n3;p1-CAGCTGTC** | **scrt2 dpn |rho** |
| **n4;p1-GAATCA** | **wor2 scrt2 |ato** | **n3;p1-GAAGTGCA** | **wor2 scrt |dpn** |
| **n4;p7-GCAAAA** | **nerfin2 hb wor |ato2 ac scrt amos rho dpn** |  |  |
